# Predicting Neoplastic Polyp in Patients With Gallbladder Polyps Using Interpretable Machine Learning Models: Retrospective Cohort Study

**DOI:** 10.1002/cam4.70739

**Published:** 2025-03-07

**Authors:** Zhaobin He, Shengbiao Yang, Jianqiang Cao, Huijie Gao, Cheng Peng

**Affiliations:** ^1^ Department of Hepatobiliary Surgery, General Surgery Qilu Hospital, Shandong University Jinan Shandong P.R. China

**Keywords:** gallbladder polyps, interpretable machine learning, neoplastic polyp, SHAP

## Abstract

**Objective:**

Gallbladder polyps (GBPs) are increasingly prevalent, with the majority being benign; however, neoplastic polyps carry a risk of malignant transformation, highlighting the importance of accurate differentiation. This study aimed to develop and validate interpretable machine learning (ML) models to accurately predict neoplastic GBPs in a retrospective cohort, identifying key features and providing model explanations using the Shapley additive explanations (SHAP) method.

**Methods:**

A total of 924 patients with GBPs who underwent cholecystectomy between January 2013 and December 2023 at Qilu Hospital of Shandong University were included. The patient characteristics, laboratory results, preoperative ultrasound findings, and postoperative pathological results were collected. The dataset was randomly split, with 80% used for model training and the remaining 20% used for model testing. This study employed nine ML algorithms to construct predictive models. Subsequently, model performance was evaluated and compared using several metrics, including the area under the receiver operating characteristic curve (AUC). Feature importance was ranked, and model interpretability was enhanced by the SHAP method.

**Results:**

*K*‐nearest neighbors, C5.0 decision tree algorithm, and gradient boosting machine models showed the highest performance, with the highest predictive efficacy for neoplastic polyps. The SHAP method revealed the top five predictors of neoplastic polyps according to the importance ranking. The polyp size was recognized as the most important predictor variable, indicating that lesions ≥ 18 mm should prompt heightened clinical surveillance and timely intervention.

**Conclusions:**

Our interpretable ML models accurately predict neoplastic polyps in GBP patients, providing guidance for treatment planning and resource allocation. The model's transparency fosters trust and understanding, empowering physicians to confidently use its predictions for improved patient care.

## Graphical abstract

This study presents a novel and interpretable approach to accurately predicting the neoplastic nature of gallbladder polyps (GBPs). We developed and validated interpretable machine learning (ML) models using data from 924 patients with GBPs who underwent cholecystectomy. Nine ML algorithms were employed, with the *K*‐nearest neighbors, C5.0 decision tree algorithm and gradient boosting machine models demonstrating the highest predictive efficacy for neoplastic polyps.

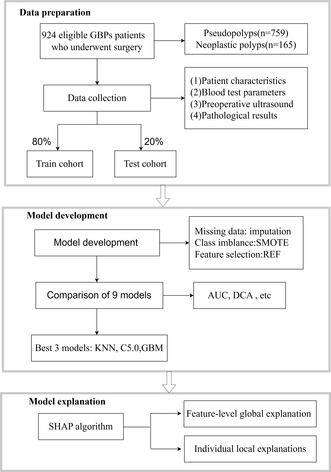

## 1 Introduction

Gallbladder polyps (GBPs) refer to a raised portion of the gallbladder's mucosal lining that protrudes into the interior of the gallbladder [1, 2]. The prevalence of GBPs is estimated to be around 5%–10% and has shown an increase in recent years, which may be primarily attributed to the widespread adoption of abdominal ultrasonography [3, 4]. At least 70% of all the GBPs are nonneoplastic (pseudopolyps), including cholesterol foci, adenomyomatosis, and inflammatory polyps, which are believed to have no potential for malignancy [5]. Only a small fraction of GBPs is neoplastic (true polyps), primarily adenomas, which are recognized to have the potential to become cancerous [6].

Patients with GBPs measuring 10 mm or larger should undergo cholecystectomy for treatment [7]. In addition to polyp size, factors such as the number of polyps, their shape, and patient age also determine the malignant potential of GBPs [5, 7, 8]. Gallbladder cancer (GBC) is characterized by an extremely poor prognosis, often due to delayed diagnosis, limited response to existing therapies, and a tendency for metastatic spread [9]. However, only a small number of GBP cases develop into GBC [10]. Therefore, a substantial number of cholecystectomies may be performed unnecessarily, underscoring the imperative for a more refined preoperative evaluation process to distinguish between neoplastic and nonneoplastic polyps [11]. Multiple studies have investigated the assessment of tumor tendency in GBPs [12, 13, 14], but accurate preoperative evaluations are lacking.

Recent advancements in machine learning (ML) and deep learning (DL) techniques have driven their widespread adoption in health care, enabling the prediction of patient outcomes through the analysis of diverse clinical data, including patient characteristics, laboratory results, and imaging findings [15, 16, 17, 18, 19]. For instance, several studies have demonstrated the utility of DL‐based models in improving the diagnostic accuracy of neoplastic GBPs using ultrasonography [20, 21, 22]. However, these studies predominantly focus on ultrasonography, while research utilizing ML or DL models to analyze clinical and pathological characteristics for differentiating neoplastic from nonneoplastic GBPs remains limited. It is worth noting that while these techniques offer powerful analytical capabilities, interpreting ML models can be challenging due to their “black‐box” nature [23]. To overcome this obstacle, the Shapley additive explanations (SHAP) method was employed to elucidate ML models and visualize predictions regarding individual variables [24].

This study aimed to create and validate interpretable ML models for precise prediction of neoplastic GBPs in a retrospective cohort, elucidate the importance of features, and explain the model using the SHAP method.

## 2 Materials and Methods

### 2.1 Study Population

The clinicopathological data of 924 patients diagnosed with GBPs who underwent cholecystectomy at Qilu Hospital of Shandong University, China, between January 2013 and December 2023 were collected. For our sample size determination, we employed the R package “pmsampsize” (version 1.1.3), which is specifically designed to compute the minimum sample size required for developing new multivariable prediction models [25]. Based on previous literature and our dataset, the prevalence of neoplastic polyps was set at 20% [12, 13]. With 36 features in our dataset and a target *C*‐statistic of 0.950 for model performance, the calculated sample size required for the study was 847. Our chosen sample size aligns with this calculated requirement. The data were extracted from the hospital's electronic medical records and pathology database. The exclusion criteria were as follows: (1) patients who were diagnosed with GBC based on postoperative pathological examination and (2) patients with more than 30% of the required variables missing. The study was approved by the Institutional Review Board of Qilu Hospital (registration number: KYLL‐2024(ZM)‐685). Due to the retrospective nature of the study, the requirement for written informed consent was waived. All study methods were conducted in accordance with the ethical principles outlined in the Declaration of Helsinki.

### 2.2 Data Collection

The data of eligible patients were obtained from the database of Qilu Hospital, Shandong University, including the following: (1) patient characteristics: age, gender, body weight, history of tobacco and alcohol use, and presence of preexisting medical conditions, including elevated blood pressure, impaired glucose metabolism, and cholelithiasis; (2) routine laboratory blood test parameters, including a complete blood count and liver function tests; (3) preoperative ultrasound, including the number of polyps, polyp size, echogenicity, and gallbladder wall characteristics; and (4) postoperative pathological results, including nonneoplastic polyps and neoplastic polyps.

### 2.3 Model Development and Validation

The enrolled patients were randomly divided into two groups using *K*‐fold cross‐validation (*k* = 5), with 80% allocated for training and 20% for internal testing, to mitigate concerns of overfitting. All selected variables contained < 30% missing values. Data were assumed missing at random and were imputed using fully conditional specification with the R “mice” package. The synthetic minority over‐sampling technique (SMOTE) was used during model training to mitigate imbalanced class distribution and optimize performance [26]. To ensure comparability among variables, all numerical predictors were scaled to be within the range of 0–1. Variance inflation factor (VIF) was calculated using the vif function from the “car” package in R to assess collinearity among the predictor variables. The recursive feature elimination (RFE), a wrapper method, iteratively evaluates and removes the least important features, identifying those most relevant for predicting the target variable and improving the model's predictive accuracy. Utilizing the rfeControl and rfe functions from the R “caret” package (version 6.0‐94), the Random Forest (rfFuncs) algorithm was implemented within the RFE framework for feature selection. Five features, including the size of the polyps, total cholesterol (CHO), gamma‐glutamyl transpeptidase (GGT), age, and hemoglobin, were incorporated into the construction of the model. For model training and hyperparameter tuning, the trainControl function from the R “caret” package was utilized. A fivefold repeated cross‐validation (repeatedcv) strategy with a 70:30 training‐to‐validation data split was implemented, and model performance was evaluated using the two‐class classification method twoClassSummary.

This study employed nine ML models to predict neoplastic polyps in GBPs, including gradient boosting machine (GBM), extreme gradient boosting (XGB), naive Bayes network (NB), multilayer perceptron (MLP), C5.0 decision tree algorithm (C5.0), Gaussian process (GP), neural network (NN), support vector machine (SVM), and *K*‐nearest neighbors (KNN).

Various commonly utilized evaluation metrics, including area under the precision–recall curve (AUC‐PR), sensitivity, specificity, F1 score, accuracy, positive predictive value (PPV), negative predictive value (NPV), and decision curve analysis (DCA), were employed to assess the reliability of these models.

The SHAP method offers insights at both the global and local levels for model interpretation. The global explanation provides consistent and precise attribution values for each feature in a model, clarifying the relationships between input features and neoplastic polyps. The local explanation demonstrates a particular prediction for an individual patient using specific input data.

### 2.4 Statistical Analysis

Statistical analyses were conducted utilizing the R statistical software (version 4.2.2, Windows platform; http://www.r‐project.org/). In addition, categorical variables were presented as absolute numbers and percentages, and the differences between groups were compared using either the chi‐squared test or Fisher's exact test (for expected frequencies < 10). Continuous variables were expressed as median and interquartile range (IQR), and the Wilcoxon rank‐sum test was employed for between‐group comparisons of two groups. In this study, the statistical significance was defined as *p* < 0.05.

## 3 Results

### 3.1 Patient Characteristics

**FIGURE 1:**
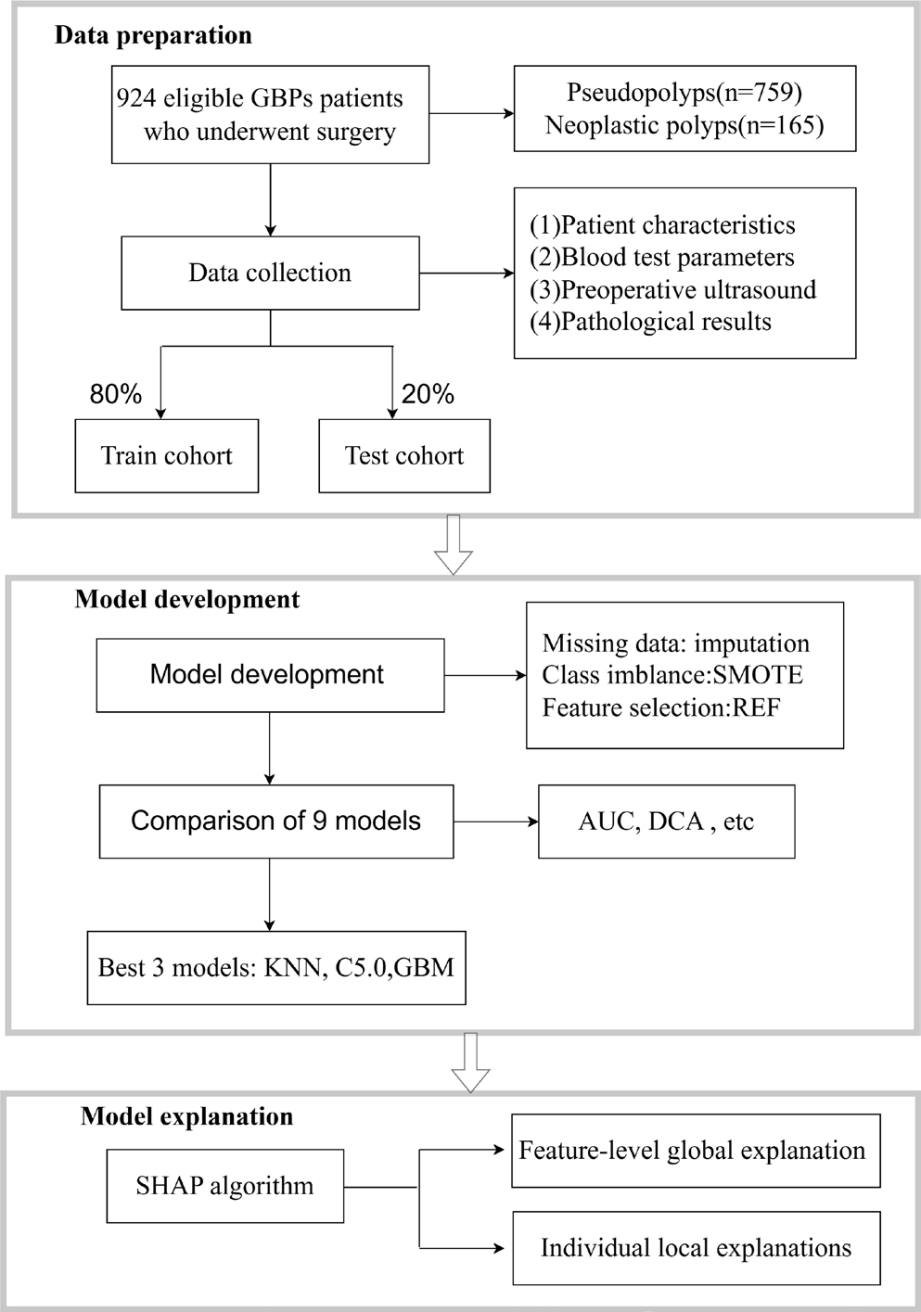
Flowchart of the study design (by Figdraw). AUC, area under the curve; C5.0, C5.0 decision; DCA, decision curve analysis; GBM, gradient boosting machine; GBPs, gallbladder polyps; REF, recursive feature elimination; SHAP, Shapley additive explanations; SMOTE, synthetic minority over‐sampling technique; XGB, extreme gradient boosting.

Figure 1 illustrates the patient screening process, describing the procedure for identifying and selecting eligible patients. The final cohort included 924 adult patients diagnosed with GBP who underwent surgery at our hospital between January 2013 and December 2023. Among the cases, 17.86% (165/924) were attributed to neoplastic polyps. The cohort was randomly split into two parts: 80% for model training and 20% for model testing. Table 1 presents the comparison of predictor variables between the training and testing cohorts.

**TABLE 1:** All predictor variables for patients with gallbladder polyps (*N* = 924).

	Pseudopolyps (*n* = 759)	Neoplastic polyps (*n* = 165)	*p*
Gender, *n* (%)			
Female	496.00 (65.00)	93.00 (57.00)	0.037
Male	263.00 (35.00)	72.00 (43.00)	
Age (years), median (IQR)	46.00 (35.00, 54.00)	46.00 (35.00, 58.00)	0.132
Weight (kg), median (IQR)	65.00 (58.00, 75.00)	65.00 (57.00, 76.00)	0.794
Smoke history, *n* (%)			
Nonsmoker	661.00 (87.00)	135.00 (82.00)	0.099
Smoker	98.00 (13.00)	30.00 (18.00)	
Drinking history			
Nondrinker	603.00 (79.00)	125.00 (76.00)	0.344
Drinker	156.00 (21.00)	40.00 (24.00)	
Hypertension, *n* (%)			
No	620.00 (82.00)	133.00 (81.00)	0.831
Yes	139.00 (18.00)	32.00 (19.00)	
Diabetes, *n* (%)			
No	722.00 (95.00)	154.00 (93.00)	0.455
Yes	37.00 (5.00)	11.00 (7.00)	
Gallstone, *n* (%)			
No	657.00 (87.00)	129.00 (78.00)	0.009
Yes	102.00 (13.00)	36.00 (22.00)	
Blood type, *n* (%)			
A	171.00 (23.00)	29.00 (18.00)	0.248
B	307.00 (40.00)	62.00 (38.00)	
AB	130.00 (17.00)	36.00 (22.00)	
O	151.00 (20.00)	38.00 (23.00)	
ALT (U/L), median (IQR)	15.00 (11.00, 22.00)	15.00 (10.00, 23.00)	0.945
AST (U/L), median (IQR)	18.00 (16.00, 21.00)	18.00 (15.00, 22.00)	0.987
GGT (U/L), median (IQR)	17.00 (12.00, 26.00)	18.00 (13.00, 30.00)	0.012
TBIL (μmol/L), median (IQR)	10.30 (7.80, 13.30)	10.00 (7.90, 13.40)	0.929
DBIL (μmol/L), median (IQR)	3.30 (2.70, 4.20)	3.40 (2.60, 4.30)	0.947
IBIL (μmol/L), median (IQR)	7.00 (5.20, 9.15)	6.60 (5.10, 9.30)	0.987
CHO (mmol/L), median (IQR)	4.43 (3.99, 5.01)	4.44 (3.88, 5.09)	0.944
TBA (μmol/L), median (IQR)	5.10 (3.30, 7.95)	4.70 (3.20, 7.00)	0.100
C1q (mmol/L), median (IQR)	169.77 (155.15, 184.95)	169.80 (151.70, 186.10)	0.577
LDH (U/L), median (IQR)	185.00 (167.61, 207.03)	189.00 (169.00, 207.00)	0.694
SA (mg/dL), median (IQR)	53.00 (49.61, 56.80)	53.80 (50.00, 58.38)	0.279
5NT (U/L), median (IQR)	4.00 (3.47, 4.30)	4.00 (3.40, 4.31)	0.584
Albumin (g/L), median (IQR)	45.10 (42.80, 47.30)	45.20 (43.30, 47.30)	0.648
Lymphocyte (×10^9^/L), median (IQR)	1.85 (1.51, 2.25)	1.88 (1.56, 2.23)	0.705
Neutrophil (×10^9^/L), median (IQR)	3.07 (2.52, 3.80)	2.94 (2.33, 3.78)	0.210
Basophil (×10^9^/L), median (IQR)	0.09 (0.05, 0.14)	0.08 (0.05, 0.15)	0.694
Eosinophil (×10^9^/L), median (IQR)	0.02 (0.01, 0.03)	0.02 (0.01, 0.04)	0.077
Monocyte (×10^9^/L), median (IQR)	0.35 (0.28, 0.44)	0.37 (0.29, 0.47)	0.085
Erythrocyte (×10^12^/L), median (IQR)	4.54 (4.28, 4.96)	4.72 (4.31, 4.98)	0.063
Hemoglobin (g/L), median (IQR)	137.00 (127.00, 149.00)	141.00 (129.00, 152.00)	0.020
Platelet (×10^9^/L), median (IQR)	236.00 (199.50, 275.50)	229.00 (194.00, 268.00)	0.356
HBsAg, *n* (%)			
Negative	700.00 (92.00)	152.00 (92.00)	1.000
Positive	59.00 (8.00)	13.00 (8.00)	
Number, *n* (%)			
Single	309.00 (41.00)	107.00 (65.00)	< 0.001
Multiple	450.00 (59.00)	58.00 (35.00)	
Size (mm), median (IQR)	10.00 (9.00, 12.00)	13.00 (11.00, 18.00)	< 0.001
Ultrasonic echo, *n* (%)			
Hyperechoic	643.00 (85.00)	129.00 (78.00)	0.052
Isoechoic	56.00 (7.00)	21.00 (13.00)	
Hypoechoic	32.00 (4.00)	11.00 (7.00)	
Mixed	28.00 (4.00)	4.00 (2.00)	
Gallbladder wall, *n* (%)			
Smooth	162.00 (21.00)	40.00 (24.00)	0.150
Rough	450.00 (59.00)	86.00 (52.00)	
Thickened	20.00 (3.00)	9.00 (5.00)	
Rough and thickened	127.00 (17.00)	30.00 (18.00)	

Abbreviations: 5NT, 5′‐nucleotidase; ALT, alanine aminotransferase; AST, aspartate aminotransferase; C1q, complement component 1q; CHO, cholesterol; DBIL, direct bilirubin; GGT, gamma‐glutamyl transferase; HBsAg, hepatitis B surface antigen; IBIL, indirect bilirubin; LDH, lactate dehydrogenase; SA, serum albumin; TBA, total bile acids; TBIL, total bilirubin.

### 3.2 Model Development and Performance Comparison

Among the nine models evaluated in the training cohorts, the KNN model demonstrated the highest predictive efficacy for neoplastic polyps, achieving an AUC of 0.985, sensitivity of 0.797, specificity of 0.985, F1 score of 0.877, accuracy of 0.904, positive predictive value (PPV) of 0.975, and negative predictive value (NPV) of 0.865. The C5.0 model followed closely, with an AUC of 0.901, sensitivity of 0.796, specificity of 0.984, F1 score of 0.877, accuracy of 0.903, PPV of 0.975, and NPV of 0.865. The GBM model also performed well, recording an AUC of 0.889, sensitivity of 0.872, specificity of 0.950, F1 score of 0.900, accuracy of 0.917, PPV of 0.930, and NPV of 0.907 (Table 2).

**TABLE 2:** Performance of each model for prediction (train cohort).

Models	AUC	Sensitivity	Specificity	F1 score	Accuracy	PPV	NPV
KNN	0.985	0.797	0.985	0.877	0.904	0.975	0.865
C5.0	0.901	0.796	0.984	0.877	0.903	0.975	0.865
GBM	0.889	0.872	0.950	0.900	0.917	0.930	0.907
XGB	0.853	0.822	0.937	0.863	0.887	0.909	0.874
NN	0.818	0.794	0.916	0.834	0.864	0.878	0.854
SVM	0.807	0.774	0.914	0.821	0.854	0.873	0.842
MLP	0.774	0.779	0.880	0.805	0.837	0.832	0.840
GP	0.741	0.699	0.882	0.754	0.803	0.818	0.794
NB	0.724	0.699	0.882	0.754	0.803	0.818	0.794

Abbreviations: AUC, area under the curve; C5.0, C5.0 decision; GBM, gradient boosting machine; GP, Gaussian process; KNN, *K*‐nearest neighbors; MLP, multilayer perceptron; NB, naive Bayes network; NN, neural network; NPV, negative predictive value; PPV, positive predictive value; SVM, support vector machine; XGB, extreme gradient boosting.

**FIGURE 2:**
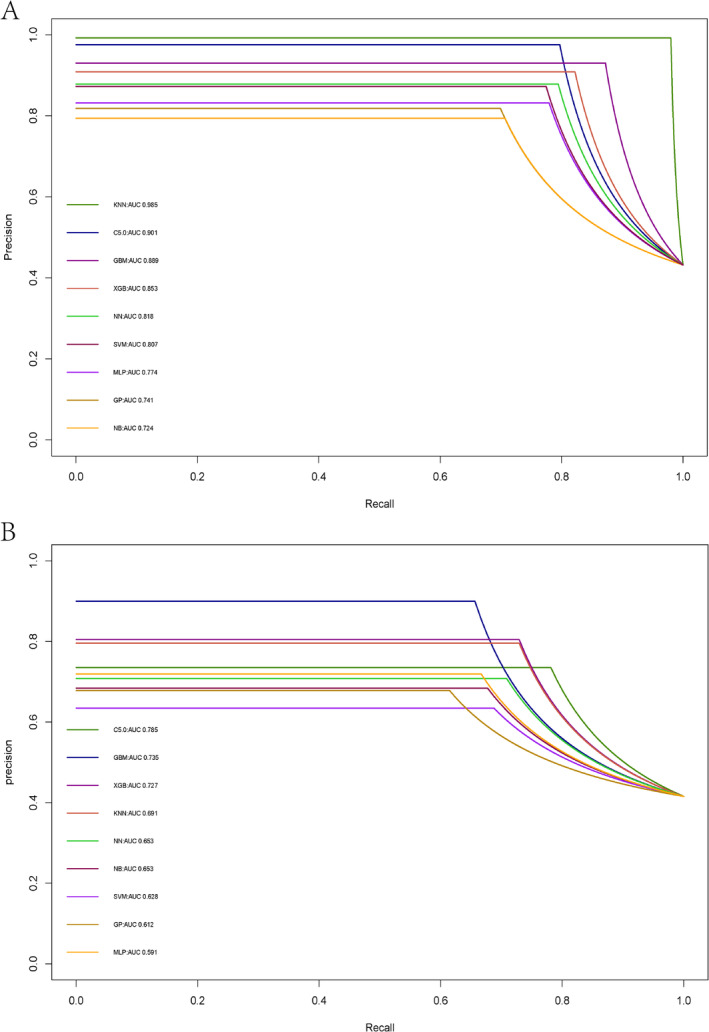
The precision–recall curve among the nine models. (A) Training cohorts; (B) testing cohorts.

**FIGURE 3:**
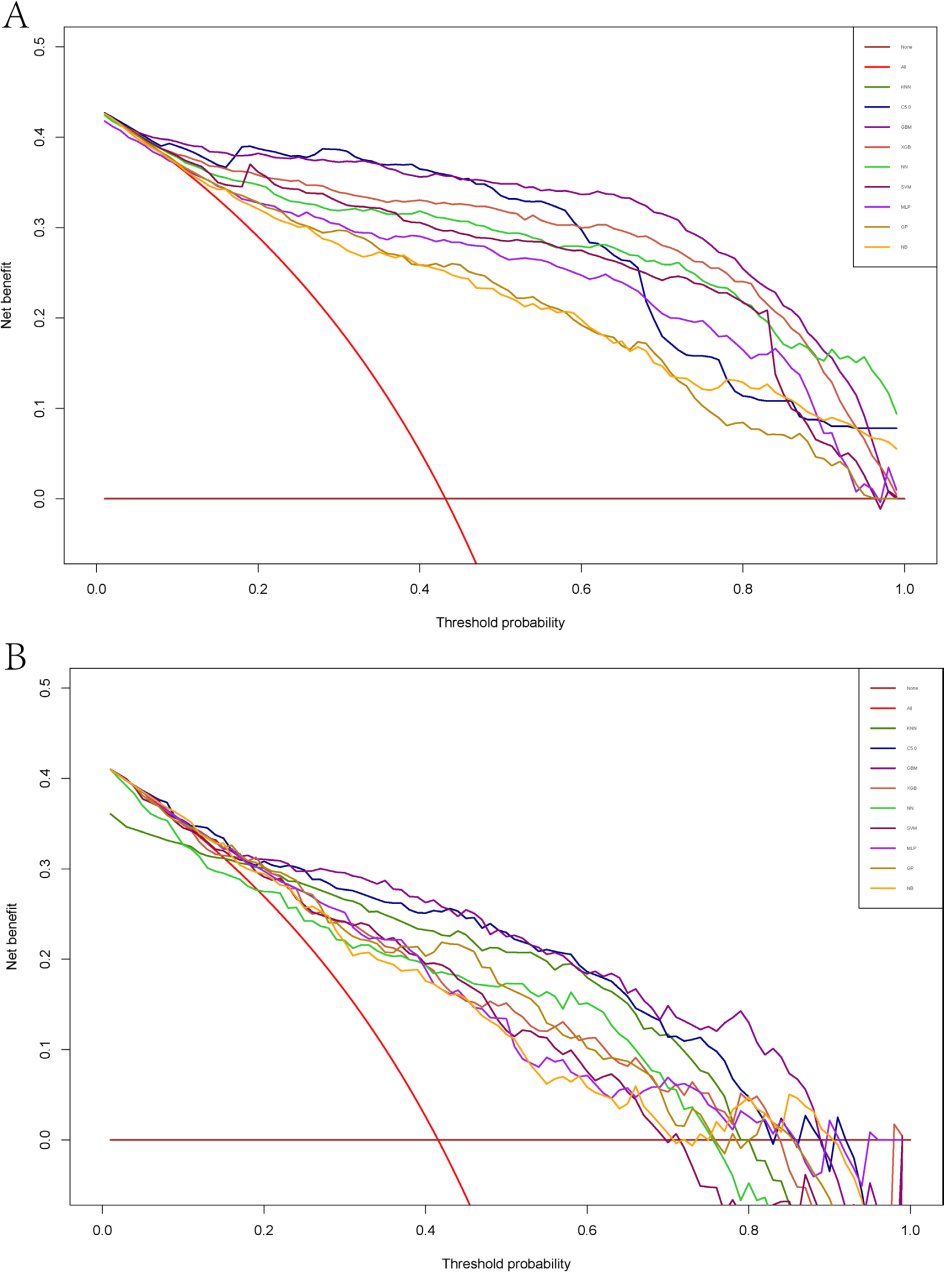
Decision curve analysis of nine models plotting the net benefit at different threshold probabilities. (A) Training cohorts; (B) testing cohorts.

In the testing cohort, three models also stood out for their performance. The KNN model achieved an AUC of 0.691, sensitivity of 0.781, specificity of 0.800, F1 score of 0.758, accuracy of 0.792, PPV of 0.753, and NPV of 0.837. Similarly, the C5.0 model demonstrated an AUC of 0.785, sensitivity of 0.656, specificity of 0.948, F1 score of 0.759, accuracy of 0.827, PPV of 0.900, and NPV of 0.795. Lastly, the GBM model achieved an AUC of 0.735, sensitivity of 0.729, specificity of 0.874, F1 score of 0.765, accuracy of 0.814, PPV of 0.805, and NPV of 0.819 (Table 3). Figure 2 displays the PR curves for both the training and testing cohorts. DCA was performed on these ML models to assess the net benefit of the best‐performing model and explore alternative strategies to guide clinical decision‐making. The GBM, C5.0, and KNN models demonstrated superior net benefits compared to other ML models (Figure 3).

**TABLE 3:** Performance of each model for prediction (test cohort).

Models	AUC	Sensitivity	Specificity	F1 score	Accuracy	PPV	NPV
KNN	0.691	0.781	0.800	0.758	0.792	0.753	0.837
C5.0	0.785	0.656	0.948	0.759	0.827	0.900	0.795
GBM	0.735	0.729	0.874	0.765	0.814	0.805	0.819
XGB	0.727	0.729	0.867	0.761	0.810	0.795	0.818
NN	0.653	0.708	0.793	0.708	0.758	0.708	0.793
SVM	0.628	0.677	0.778	0.681	0.736	0.684	0.772
MLP	0.591	0.688	0.719	0.660	0.706	0.635	0.764
GP	0.612	0.615	0.793	0.645	0.719	0.678	0.743
NB	0.653	0.667	0.815	0.692	0.753	0.719	0.775

### 3.3 Model Explanation

**FIGURE 4:**
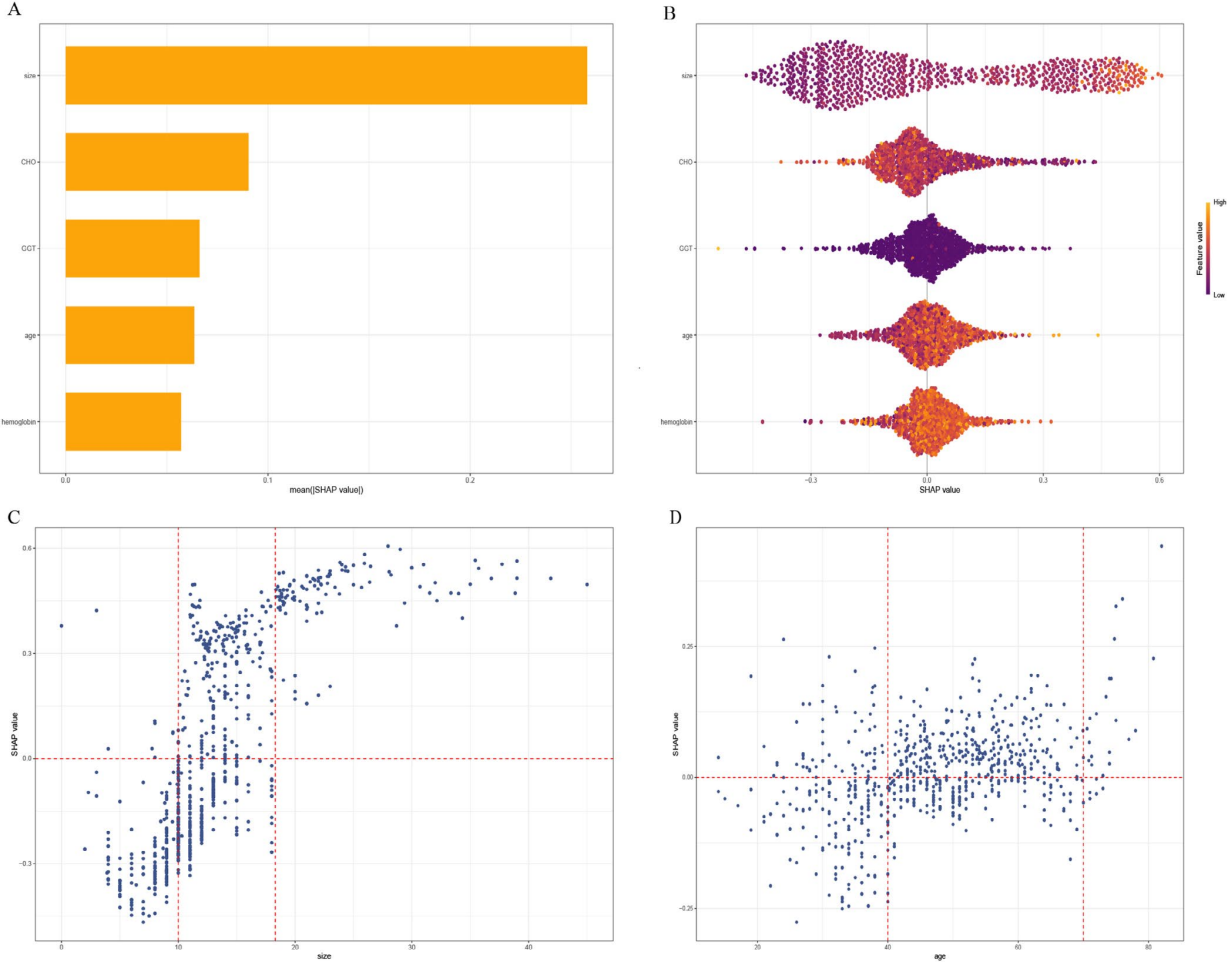
Global model explanation by the SHAP method. (A) SHAP summary bar plot; (B) SHAP summary dot plot (neoplastic polyp probability increases with feature SHAP values. Each patient has a dot on the line for each feature, colored by feature value. The dot density shows feature value distribution); (C, D) SHAP dependence plot (each dependence plot illustrates the influence of an individual feature on the output of the prediction model, with each data point representing a distinct patient).

**FIGURE 5:**
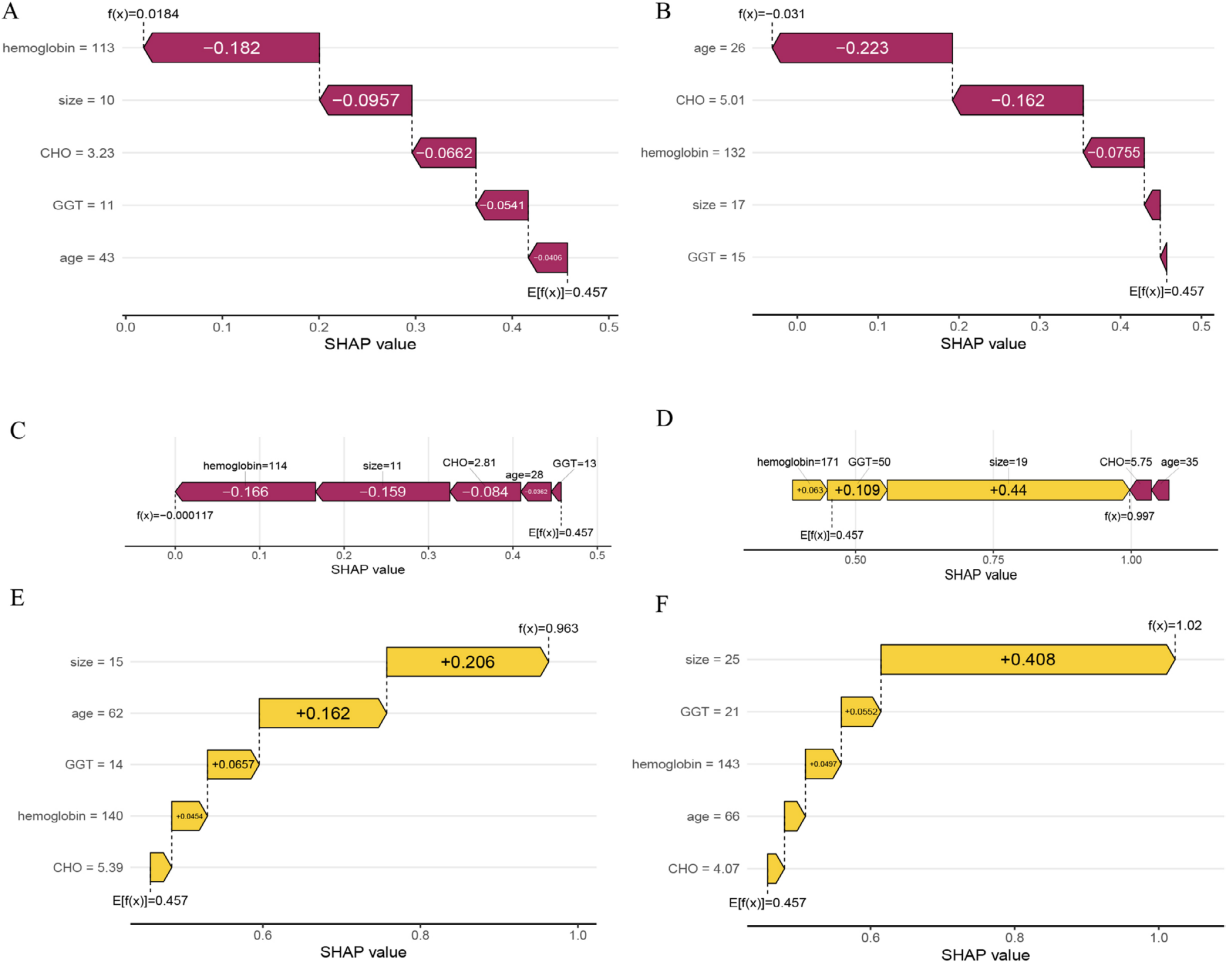
Local model explanation by the SHAP method. (A–C) The individual patient toward the “pseudopolyps” class; (D–F) The individual patient toward the “neoplastic polyp” class.

The SHAP algorithm was employed to assess the importance of each predictor variable in relation to the predicted outcome of the final model. This methodology provided two types of explanations, namely a feature‐level global explanation of the model's performance and individualized local explanations tailored to specific instances. The global explanation provided an overview of the model's overall functionality. Figure 4A,B illustrates the evaluation of feature contributions to the model through average SHAP values, presented in descending order. Across all prediction horizons, the size of the polyps presented the highest average SHAP value, exceeding that of other predictors by more than double. Other important predictors included CHO, GGT, age, and hemoglobin. Additionally, the SHAP dependence plot provided valuable insights into the relationship between individual features and the predictions generated by the model. Figure 4C,D illustrates the relationship between the actual values and the corresponding SHAP values for the five selected features. SHAP values exceeding zero indicate that the model predicts a higher probability of the positive class, suggesting an increased likelihood of neoplastic polyps. In this study, polyp size was found to strongly influence the model's results, with sizes ≥ 18 and ≤ 10 mm having positive and negative SHAP values, respectively. These findings supported the decision toward the “neoplastic polyp” and “nonneoplastic polyp” classes. The impact of polyp size on SHAP values was more prominent compared to other predictors, which showed greater uncertainty. These results highlight the dominant role of polyp size in the model's decision‐making process and the substantial heterogeneity among individual patients. Furthermore, the local explanation provided insights into how the model generated a specific prediction for an individual patient by considering their unique input data (Figure 5).

## 4 Discussion

To the best of our knowledge, this is the first retrospective study to examine and compare nine ML models for the analysis of neoplastic polyp prediction in GBP cohorts. This study delineated a cluster of predictive risk factors, demonstrating exceptional performance across the evaluated models. These models analyzed routine and readily accessible preoperative clinical data, offering convenient and valuable insights to guide treatment.

Several prior studies have utilized the logistic regression algorithm to develop nomograms for predicting neoplastic polyps [12, 13, 14]. Age, carcinoembryonic antigen, polyp size, and sessile shape have been identified as independent predictors of the neoplastic potential of GBPs. However, relying on a singular analyte in clinical practice can pose challenges due to the heterogeneity of patients. ML and DL techniques excel in handling complex and extensive data by analyzing and training on highly variable datasets and complex variable relationships. Currently, the literature based on DL for predicting neoplastic polyps predominantly addresses ultrasonography radiomics, lacking a comprehensive focus on patients' clinical and pathological characteristics [20, 21].

Among the nine ML models used in this study, the KNN, C5.0, and GBM models exhibited the highest AUC values in both the training and validation cohorts. These models also demonstrated favorable net benefits, indicating their effectiveness in predicting outcomes. The KNN algorithm is a nonparametric method for classification and regression that assigns labels based on the majority class or average of KNN, with performance influenced by the choice of *k* and distance metric, but it is computationally expensive and sensitive to irrelevant features [27]. C5.0 is an advanced decision tree algorithm for classification that recursively partitions data based on informative features, but it suffers from overfitting, noise sensitivity, and bias toward features with many categories, which can be mitigated through pruning, ensemble methods, data preprocessing, and cross‐validation [28, 29]. GBM is a powerful ensemble learning algorithm that iteratively combines weak learners to create a strong predictive model, capturing complex patterns in data while offering flexibility in tuning and interpretation [30].

In clinical practice, a GBP size of ≥ 1 cm is widely used as an arbitrary criterion to determine the need for cholecystectomy [8, 31, 32, 33, 34]. However, neoplastic polyps tend to be larger, with a mean size ranging from 18 to 21 mm, compared to nonneoplastic polyps, which have a mean size ranging from 4 to 7.5 mm. Therefore, the 1 cm surgical threshold may not be adequate to indicate the need for surgery [35, 36]. The SHAP framework was employed to interpret the XGBoost model, and several key variables (polyp size, CHO, GGT, age, and hemoglobin) were found to significantly contribute to the prediction of neoplastic polyps. Consistent with previous studies, polyps larger than 18 mm were considered to be potentially neoplastic polyps. Notably, the predominant influence of polyp size as a predictor for the neoplastic potential of GBPs significantly outweighed that of other predictors. Nonetheless, literature regarding several metabolic predictor factors within the model remains scarce. For instance, previous literature suggests that dyslipidaemia is associated with the formation of GBPs [37, 38, 39, 40]. However, the relationship between cholesterol levels and both GBPs and GBC remains ambiguous, highlighting the need for further investigation.

Nevertheless, the limitations of the present study should be acknowledged. First, due to the retrospective design of this study, potential selection bias could not be completely eliminated. For example, there is missing data for certain preoperative ultrasound characteristics of the polyps, such as the sessile shape of the polyps. Second, although ML techniques allow the construction of predictive models with internal validation, the results lack external validation and were derived from a relatively small sample size from a single center. Third, the study may also be limited by the class imbalance in the data [41, 42], which was mitigated through the use of the SMOTE oversampling technique [26, 43, 44]. Fourth, applying ML models in clinical practice presents challenges. The models should seamlessly integrate with existing electronic health record (EHR) systems to facilitate smooth access and data flow. While cloud‐based solutions can offer the necessary computational resources, they necessitate reliable internet connectivity and robust cybersecurity measures. Additional challenges may include the need for clinician training and overcoming resistance to change, as well as addressing regulatory compliance and data privacy concerns. Ultimately, if implemented effectively, the model has the potential to enhance clinical workflows by delivering timely insights, improving decision‐making processes, and positively influencing patient outcomes. An interesting example is the development of a web application where relevant features can be input to produce predictive outcomes [45]. However, this approach requires adequate supporting resources.

## 5 Conclusions

Interpretable ML models were developed to accurately predict neoplastic polyps in patients with GBPs by analyzing clinical data and ultrasound imaging features. Polyp size was the most robust predictor of neoplastic GBPs, with lesions measuring ≥ 18 mm warranting heightened clinical surveillance and prompt intervention to facilitate early cancer detection and optimize patient outcomes.

## Author Contributions

**Zhaobin He:** conceptualization (equal), data curation (lead), formal analysis (lead), writing – original draft (lead). **Shengbiao Yang:** data curation (equal), formal analysis (equal). **Jianqiang Cao:** data curation (equal). **Huijie Gao:** data curation (equal). **Cheng Peng:** conceptualization (equal), supervision (lead), writing – review and editing (lead).

## Ethics Statement

The study was approved by the Institutional Review Board of Qilu Hospital (registration number: KYLL‐2024(ZM)‐685). Due to the retrospective nature of the study, the requirement for written informed consent was waived. All study methods were conducted in accordance with the ethical principles outlined in the Declaration of Helsinki.

## Conflicts of Interest

The authors declare no conflicts of interest.

## Data Availability

The data used and/or analyzed during the current study are available from the corresponding author upon reasonable request.
